# Transforaminal lumbar interbody fusion with or without release of the anterior longitudinal ligament: A single-center, retrospective observational cohort study

**DOI:** 10.1016/j.xnsj.2024.100533

**Published:** 2024-07-29

**Authors:** Samantha Högl-Roy, Nader Hejrati, Felix C. Stengel, Stefan Motov, Anand Veeravagu, Benjamin Martens, Martin N. Stienen

**Affiliations:** a Spine Center of Eastern Switzerland, Kantonsspital St. Gallen & St. Gallen Medical School, Rorschacher Str. 95, St. Gallen 9000, Switzerland; b Department of Neurosurgery, Kantonsspital St. Gallen & St. Gallen Medical School, Rorschacher Str. 95, St. Gallen 9000, Switzerland; c Department of Neurosurgery, School of Medicine, Stanford University, 453 Quarry Road, Palo Alto, CA 94304, United States; d Department of Orthopedic Surgery, Kantonsspital St. Gallen & St. Gallen Medical School, Rorschacher Str. 95, St. Gallen 9000, Switzerland

**Keywords:** Transforaminal lumbar interbody fusion, Anterior longitudinal ligament, Anterior release, Spinal deformity, Sagittal balance, Complications, Outcome

## Abstract

**Background:**

Transforaminal anterior release (TFAR) is a technical extension of the transforaminal lumbar interbody fusion (TLIF) procedure with deliberate release of the anterior longitudinal ligament (ALL).

**Methods:**

In a retrospective, single-center observational cohort study, consecutive adult patients undergoing TLIF surgery at L4/L5 and/or L5/S1 between 01/2018 and 12/2022 for degenerative disc disease or deformity were considered. The TFAR group (with ALL release) was compared to a standard TLIF group (without ALL release), matched in a 1:3 ratio. Uni- and multivariable logistic regression models were built to estimate the likelihood of any adverse event (AE), reoperation, and excellent/good clinical outcome at 12 months.

**Results:**

Of 438 patients, 18 undergoing TFAR were matched to 53 undergoing standard TLIF. TFAR procedures were frequently part of extensive, anterior-posterior or multilevel fusion procedures with longer surgery time and higher blood loss. The rates of intraoperative surgical AEs were similar (16.7 vs. 11.3%, p=.789). The rates and severities of surgical AEs, as well as reoperation rates and clinical outcomes were similar at time of discharge, 90 days, and 12 months postoperatively (all p>.05). TFAR allowed for an increase in total lumbar lordosis of 16.1° and in lumbar lordosis between L4 and S1 of 16.3° at discharge, which was maintained during follow-up. In both the uni- and multivariable models, patients undergoing TFAR were as likely as patients undergoing standard TLIF to experience any AE (adjusted OR 0.78, 95% CI 0.21–2.94), any reoperation (aOR 0.46, 95% CI 0.11–1.90) or excellent/good clinical outcome at 12 months (aOR 2.01, 95% CI 0.52–7.74).

**Conclusions:**

The TFAR technique has a safety profile which is comparable to the standard TLIF procedure, but it allows for a greater restoration of lumbar lordosis at L4-S1. We suggest considering the TFAR technique in selected patients with sagittal imbalance and mobile segments for restoration of lumbar lordosis.

## Introduction

Transforaminal lumbar interbody fusion (TLIF) is an established surgical procedure for improving the quality of life of patients suffering from a range of degenerative diseases of the lumbar spine [1,2]. Recently, a technological extension of the TLIF was described by Sweet and Sweet [3], aiming at increasing the segmental and overall lumbar lordosis by controlled release of the anterior longitudinal ligament (ALL) through the posterior approach. This so-called transforaminal anterior release (TFAR) is, however, a more demanding procedure that allows for a greater correction of the sagittal imbalance.

With an increasing number of reports describing the TFAR technique, it becomes evident that this technique can be considered safe and effective [[4], [5], [6]]. However, some groups have used the TFAR technique exclusively or preferably in the upper lumbar spine [4,6], whereas in patients with Roussouly type 1 to 3 geometry, most of the lumbar lordosis should be restored in the lower lumbar spine, between L4 and S1 [7]. The usually more advanced degenerative changes in the lower lumbar spine and its association with a deeper surgical site as well as close proximity between the disc spaces and the vascular structures anterior to the spine render a remobilization and restoration of impaired sagittal balance in the lower lumbar spine more challenging. As such, to date, there are only few reports on TFAR between L4 and S1 [3,5].

We here set out to review our series of patients treated by the TFAR technique between L4 and S1. In this study, it was our aim to provide more data from another center applying this novel technique, and to fill the knowledge gap by directly comparing patients who underwent TLIF with versus without ALL release, focusing on spino-pelvic parameters, complications, and overall outcome until 1 year postoperatively.

## Material and methods

### Patient identification, in- and exclusion criteria

We conducted as a single-center, retrospective cohort study, reviewing operative notes of consecutive adult patients who underwent spinal fusion surgery including a TLIF procedure in the lumbar spine at the levels L4/5 or L5/S1 for degenerative disc disease or deformity, at the Spine Center of Eastern Switzerland, Kantonsspital St.Gallen, between 01/2018 and 12/2022 with completed 1-year follow-up. Operative reports were carefully reviewed to identify cases, where the ALL was intentionally released (TFAR); these patients were considered the study group. A control group with 3 times the number of randomly chosen patients who underwent a TLIF procedure without ALL release during the same time interval was built.

We only included patients who signed the institutional general consent to allow de-identified research, with sufficient clinical and radiological data available prior and after the surgery. Exclusion criteria were (1) other surgical indication (tumor, infection, trauma), (2) TLIF performed at any other level than L4/L5 and L5/S1 or (3) TLIF performed for reposition of isthmic spondylolisthesis.

### Ethical considerations

The institutional review board (IRB) of St.Gallen approved the study (BASEC 2023-01343). Retrospective collection, analysis and publication of anonymized patient data was allowed with an institutional waiver for informed consent.

### Indication and surgical treatment

**Fig. 1:**
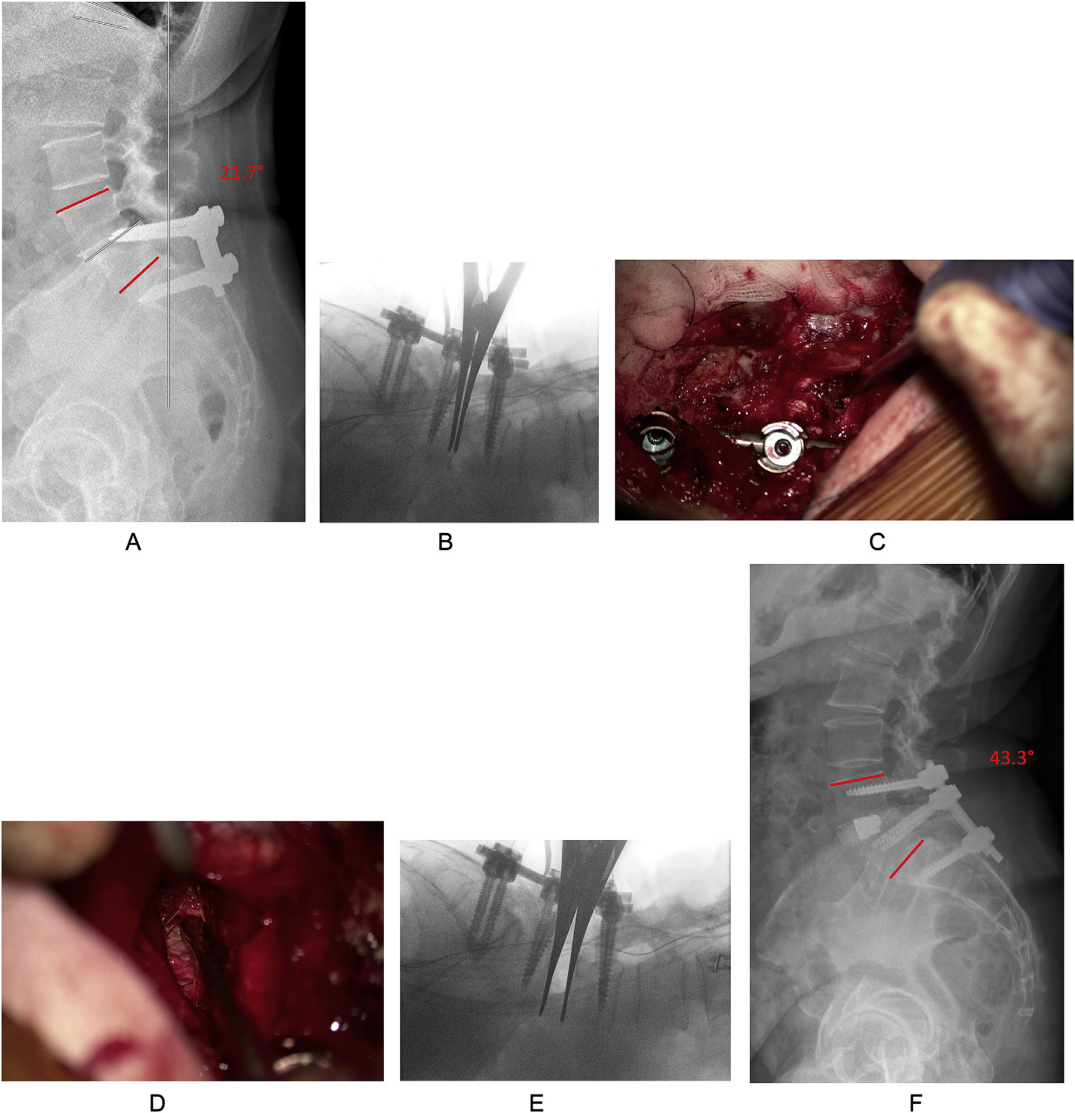
This 63-year-old female with history of instrumented fusion at L5/S1 some 30 years prior was referred to our spine center with immobilizing low back pain and bilateral, radicular lower extremity pain (VAS back 7/10, VAS leg 7/10, Oswestry Disability Index (ODI) 26.7/100%, Core Outcome Measures Index (COMI) Back score 6.9/10) owing to adjacent segment disease with spinal stenosis and failed extended conservative treatment. There are anatomical peculiarities with a singular pelvic kidney ventral to the lumbar spine and tortuous arterial vessels between the psoas muscle and the discal compartment at the L4/5 level, rendering both an anterior and lateral approach to the L4/5 segment unsafe. (A) Preoperative standing x-ray, showing the misplaced L5 screws violating the L4/5 disc space with degenerative spondylolisthesis. Her pelvic incidence is 69° (Roussouly type-4 geometry), requiring a total lumbar lordosis (LL) of 65° and LL of 39°–42° between L4 and S1. Her actual lordosis between L4 and S1 was 21.7° (illustrated in red). (B) After screw placement and bilateral facetectomy the spondylolisthesis reduction is performed, but the segment cannot be opened widely at this time. C: The 4mm chisel is taken to perforate the ALL in a safe region without proximity to blood vessels (Video 1). (D) Then, the ALL is resected from both sides with the 2 mm Kerrison punch until retroperitoneal fat is visualized (Video 2). (E) Now, the segment gets very mobile and can be opened as wide as desired with the Chiari interbody spreader. (F) After graft insertion and placement of the cage, posterior compression is applied until the desired degree of segmental lordosis is restored. In this patient, the 3-month postoperative x-ray shows restoration of 43.3° of LL between L4 and S1 (illustrated in red). The patient experienced no complications and was recovering well (VAS back 0/10; VAS leg 0/10; ODI 13.3/100%; COMI Back score 3.0/10).

The indications for standard TLIF were degenerative disc disease (DDD) with spondylolisthesis, foraminal stenosis, segmental instability and/or facet joint disease. TLIFs were extended to TFAR in patients with severe lack of segmental lordosis (SL), in which anterior or lateral approaches (ALIF, LLIF/XLIF/OLIF) were not possible for reasons including patient-specific anatomy (Fig. 1), previous abdominal/retroperitoneal surgeries, or ankylosed facet joints requiring posterior release prior to the anterior approach.

Our standard TLIF procedure includes placement of pedicle screws first to provide initial posterior rod-based segmental distraction after resection of the inferior articulating processes (Fig. 1A and B). After reduction of the superior articulating process, the disc space is incised within the Cambin's triangle and a thorough discectomy is conducted, followed by meticulous endplate preparation. The disc space is opened as wide as possible using chisels to allow for wide access to the disc and introduce a modified Chiari spreader for interbody distraction (Fig. 1B). In patients with sagittal deformity and in all TFAR cases, bilateral complete facetectomies (Schwab type II) are conducted and the discectomy is conducted from both sides [8]. At some point tension from the ALL prevents further distraction of the disc space using the interbody spreader, therefore, set screws are tightened to maintain the distraction. With the TFAR technique, the segment is now released by introducing a 4 or 8mm chisel and gentle palmar taps of the hands to cut the lateral annulus first, followed by the ALL (Fig. 1C; Video 1). In patients where there is no fat/distance between the disc and the inferior vena cava or common iliac vein as seen on preoperative imaging, cutting this part of the annulus/ALL is omitted and instead will gently open with further distraction once the remaining two thirds have been released. If the anatomy does not allow for sufficient visualization of the anterior aspects of the disc space, we use the microscope and a 2mm Kerrison punch to safely release the ALL and to visualize the retroperitoneal fat (Fig. 1D). After checking for a sufficient anterior release (Video 2), the interbody spreader is reinserted into the disc space, and the segment can be opened much wider now (Fig. 1E). Once the trial implant(s) have been sized, bone graft is placed into the disc space, followed by the definitive interbody cage, which is brought into its final position under serial x-ray imaging. We position the cages sufficiently anterior, but keep a safety distance from the most anterior aspect of the vertebral body to avoid cage migration into the retroperitoneal space [9]. In some cases we have used FibreWire high strength surgical sutures (Arthrex GmbH, Munich, Germany) to temporarily secure the interbody cage while positioning it into the anterior aspect of the intervertebral space in order to prevent migration into the retroperitoneal space (the wire was again removed after final cage position was obtained). Expandable interbody spacers were opened until the desired height was reached and finally graft material was applied to fill up the posterior aspect of the interbody space. Posterior compression over the screw heads is then conducted until the desired degree of SL is obtained (Fig. 1F).

### Variables and data collection

All patient data were entered into electronic case report forms included in a SecuTrial database, run by the Clinical Trials Unit of our hospital. We collected data at 5 different time points: (1) last preoperative outpatient consultation, (2) hospital admission for surgery, (3) surgery, (4) outpatient consultation as close as possible to 90 days (mean 79.6 days, SD 22.7) and (5) outpatient consultation as close as possible to 1 year postoperatively (mean 357.5 days, SD 103.8).

The database contains demographic (e.g., age, body mass index, smoking status, disease type, previous spine surgeries), surgical, complication, and outcome variables included in the Eurospine Spine Tango registry [10], but was extended to capture the spino-pelvic parameters before and after surgery in more detail. Moreover, complications were assessed and their severity was graded by the novel Therapy-Disability-Neurology scale [11]. As patient-reported outcome measures (PROMS) were only introduced in our department in 01/2022, clinical outcome was graded according to the MacNab criteria into 4 categories (excellent, good, fair, poor), as best estimated from the discharge or outpatient consultation letter [12].

### Statistical considerations

Our independent variable of interest was TFAR; patients with TFAR were assigned to the study group and those with a standard TLIF procedure were assigned to the control group. Normality of data was tested using Shapiro Wilk tests. First, baseline demographic and surgical parameters were compared using ranks sum and chi-square tests (Tables 1 and 2), as appropriate. Then, intra-/postoperative complications and outcomes were compared between the groups, illustrated in Table 2, Table 3, Table 4, again using ranks sum and chi-square tests, as appropriate. Patient's spino-pelvic parameters were analyzed in detail in Table 5, using 2-sided t-tests, rank sum and chi-square tests to compare the values or categories at each time point. As we had noticed some differences between the study and control group in terms of age, history of repeat surgery, level of TLIF and number of segments included in the fusion procedure, a logistic regression model was built to estimate the influence of TFAR on 3 main outcomes (Table 6): (1) any complication until 12 months postoperatively, (2) any reoperation until 12 months postoperatively, and (3) excellent or good outcome at 12 months. In a multivariable logistic regression model, we calculated adjusted odds ratios (ORs) and 95% confidence intervals (95% CIs), independent of these potential confounders.

**Table 1:** Baseline demographic and disease-specific information on n=71 patients with degenerative disc disease undergoing standard TLIF surgery, or TLIF with release of the anterior longitudinal ligament (ALL) between L4 and S1 (transforaminal anterior release = TFAR).

	TFAR	Standard TLIF	p-value
Age, in y	64.2 (10.0)	68.4 (10.8)	.074
Sex			.455
Female	11 (61.1%)	27 (50.9%)	
Male	7 (38.9%)	26 (49.1%)	
Body mass index, in kg/m^2^	28.3 (5.7)	27.2 (6.3)	.707
Smoking status			.724
Nonsmoker	14 (77.8%)	39 (73.6%)	
Smoker	4 (22.2%)	14 (26.4%)	
ASA grade			.263
I – no morbidity	1 (5.6%)	1 (1.9%)	
II – mild/moderate	8 (44.4%)	30 (56.6%)	
III – severe	8 (44.4%)	22 (41.5%)	
IV – life threatening	1 (5.6%)	−(0.0%)	
Work status			.607
Not working (invalidity)	1 (5.6%)	2 (3.8%)	
Not working (retired)	10 (55.6%)	37 (69.8%)	
Not working (sick leave)	2 (11.1%)	2 (3.8%)	
Working, full job	5 (27.8%)	9 (17.0%)	
Working, partial job	−(0.0%)	2 (3.8%)	
Missing data	−(0.0%)	1 (1.9%)	
Main pathology			.133
Spinal stenosis	4 (22.2%)	19 (35.9%)	
Spondylolisthesis	2 (11.1%)	13 (24.5%)	
Deformity	12 (66.7%)	21 (39.6%)	
Preoperative symptoms			
Axial low back pain	18 (100%)	52 (98.1%)	.557
Peripheral radiating pain	18 (100%)	52 (98.1%)	.557
Motor deficit	2 (11.1%)	1 (1.9%)	.093
Sensory deficit / dysesthesia	17 (94.4%)	49 (92.5%)	.775
Bowel/bladder dysfunction	−(0.0%)	1 (1.9%)	.557
Functional status			.775
Excellent	−(0.0%)	−(0.0%)	
Good	−(0.0%)	−(0.0%)	
Fair	1 (5.6%)	4 (7.6%)	
Poor	17 (94.4%)	49 (92.5%)	
Pain medication			
NSAID/Paracetamol (WHO I)	10 (55.6%)	29 (54.7%)	.951
Weak opioids (WHO II)	1 (5.6%)	4 (7.5%)	.775
Strong opiates (WHO III)	6 (33.3%)	13 (24.5%)	.466
Admission type			.775
Elective	17 (94.4%)	49 (92.5%)	
Emergency	1 (5.6%)	4 (7.5%)	
Repeat surgery			.055
No	11 (61.1%)	44 (83.0%)	
Yes	7 (38.9%)	9 (17.0%)	
Previous surgeries, same segment	1.4 (1.2)	2.0 (1.4)	.707
Level of TLIF			.004
L4/5	6 (33.3%)	38 (71.7%)	
L5/S1	12 (66.7%)	15 (28.3%)	
**Total**	**n=18 (100%)**	**n=53 (100%)**	

Data is presented as count (percent) or mean (standard deviation).

**Table 2:** Surgery-specific information on n=71 patients with degenerative disc disease undergoing standard TLIF surgery, or TLIF with release of the anterior longitudinal ligament (ALL) between L4 and S1 (transforaminal anterior release = TFAR).

	TFAR	Standard TLIF	p-value
Surgeon board-certified in			.411
Neurosurgery	8 (44.4%)	23 (43.4%)	
Orthopedic surgery	10 (55.6%)	30 (57.7%)	
Surgeon's level of experience			.761
Junior attending	3 (16.7%)	13 (24.5%)	
Senior attending	7 (38.9%)	19 (35.8%)	
Leading surgeon	8 (44.4%)	21 (39.6%)	
Extent of procedure			.034
Monosegmental fusion	9 (50.0%)	31 (58.5%)	
Bisegmental fusion	4 (22.2%)	19 (35.9%)	
Multilevel fusion	5 (27.8%)	3 (5.7%)	
Anterior-posterior approach w/ LLIF/XLIF			.159
No	14 (77.8%)	48 (90.6%)	
Yes	4 (22.2%)	5 (9.4%)	
Posterior approach			.758
Midline, conventional open	17 (94.4%)	50 (96.2%)	
Transmuscular, “Wiltse”	1 (5.6%)	2 (3.8%)	
Pedicle screw placement			.047
Free-hand technique	16 (88.9%)	34 (64.2%)	
Intraoperative 3D imaging and navigation	2 (11.1%)	19 (35.8%)	
Cement-augmentation of pedicle screws			.229
No	13 (72.2%)	45 (84.9%)	
Yes	5 (27.8%)	8 (15.1%)	
Use of intraoperative neuromonitoring			.435
No	16 (88.9%)	50 (94.3%)	
Yes	2 (11.1%)	3 (5.7%)	
Length of surgery, in min	442 (163)	319 (142)	.001
Estimated blood loss, in ml	1385 (969)	581 (638)	.025
Intraoperative use of blood products			.001
None	13 (72.2%)	52 (98.1%)	
Cellsaver and retransfusion	4 (22.2%)	−(0.0%)	
Red blood cell transfusion	1 (5.6%)	1 (1.9%)	
Intraoperative surgical AEs			.789
None	15 (83.3%)	47 (88.7%)	
Dural leak	2 (11.1%)	3 (5.7%)	
Vascular injury	−(0.0%)	1 (1.9%)	
Other*	1 (5.6%)	2 (3.8%)	
Intraoperative medical AEs			.158
None	15 (83.3%)	50 (94.3%)	
Cardiovascular decompensation	2 (11.1%)	3 (5.7%)	
Other anesthesiological	1 (5.6%)	−(0.0%)	
**Total**	**n=18 (100%)**	**n=53 (100%)**	

⁎ Other listed AEs were pedicle screw loosening with implant replacement (n=1) and potential endplate violation (n=2). Data is presented as count (percent) or mean (standard deviation).

**Table 3:** Information on adverse events (AEs) and outcome at discharge in=71 patients with degenerative disc disease undergoing standard TLIF surgery, or TLIF with release of the anterior longitudinal ligament (ALL) between L4 and S1 (transforaminal anterior release = TFAR).

	TFAR	Standard TLIF	p-value
Any postoperative AE			.530
No	9 (50.0%)	31 (58.5%)	
Yes,	9 (50.0%)	22 (41.5%)	
Of those, medical complications	6 (66.7%)	15 (68.2%)	.935
Of those, surgical complications	3 (33.3%)	7 (31.8%)	
Medical AEs			.641
Cardiovascular	2 (33.3%)	5 (33.3%)	
Kidney/urinary	1 (16.7%)	2 (13.3%)	
Liver/gastrointestinal	−(0.0%)	1 (6.7%)	
Pulmonary	2 (33.3%)	2 (13.3%)	
Thromboembolism	1 (16.7%)	1 (6.7%)	
Other*	−(0.0%)	4 (26.7%)	
Surgical AEs			.337
CSF leak/pseudomeningocele	1 (33.3%)	−(0.0%)	
Implant failure	−(0.0%)	1 (14.3%)	
Implant malposition	−(0.0%)	1 (14.3%)	
New radiculopathy	−(0.0%)	1 (14.3%)	
New sensory dysfunction	−(0.0%)	2 (28.6%)	
Wound infection	1 (33.3%)	−(0.0%)	
Other†	1 (33.3%)	2 (28.6%)	
Therapy-Disability-Neurology grade of AE			.524
1 (mild AE)	1 (11.1%)	7 (31.8%)	
2 (mild to moderate AE)	5 (55.6%)	7 (31.8%)	
3 (moderate AE)	2 (22.2%)	4 (18.2%)	
4 (severe AE)	1 (11.1%)	4 (18.2%)	
5 (death)	−(0.0%)	−(0.0%)	
Unplanned reoperation			.435
No	16 (88.9%)	50 (94.3%)	
Yes, for the following reason	2 (11.1%)	3 (5.7%)	
Hardware malposition	−(0.0%)	2 (3.8%)	.230
Wound infection	1 (5.6%)	1 (1.9%)	
CSF leak	1 (5.6%)	−(0.0%)	
Intensive care unit stay			.033
Not required	8 (44.4%)	42 (79.3%)	
1 d	6 (33.3%)	5 (9.4%)	
2 d	2 (11.1%)	2 (3.8%)	
3 d	−(0.0%)	2 (3.8%)	
>3 d	2 (11.1%)	2 (3.8%)	
Discharge disposition			.641
Home and outpatient rehabilitation	9 (50.0%)	31 (58.5%)	
Inpatient rehabilitation	8 (44.4%)	21 (39.6%)	
In-hospital transfer to different unit	1 (5.6%)	1 (1.9%)	
Clinical outcome			.502
Excellent	−(0.0%)	4 (7.6%)	
Good	16 (88.9%)	40 (75.5%)	
Fair	1 (5.6%)	7 (13.2%)	
Poor	1 (5.6%)	1 (1.9%)	
Missing	−(0.0%)	1 (1.1%)	
**Total**	**n=18 (100%)**	**n=53 (100%)**	

⁎ Other listed medical AEs were anemia (n=3) and unspecific inflammatory condition (n=1).

† Other surgical AEs were unusual pain exacerbation (n=1), hemorrhagic surgical wound (n=1) and gluteal compartment syndrome (n=1). Data is presented as count (percent) or mean (standard deviation).

**Table 4:** Information on surgical adverse events (AEs) and outcome at 90 days and 12 months in=71 patients with degenerative disc disease undergoing standard TLIF surgery, or TLIF with release of the anterior longitudinal ligament (ALL) between L4 and S1 (transforaminal anterior release = TFAR).

	90 d			12 mo		
	TFAR	Standard TLIF	p-value	TFAR	Standard TLIF	p-value
Any surgical AE at 90 d			.671			.213
No	12 (66.7%)	36 (67.9%)		16 (88.9%)	40 (75.5%)	
Yes	6 (33.3%)	15 (28.3%)		1 (5.6%)	12 (22.6%)	
Missing	−(0.0%)	2 (3.8%)		1 (5.6%)	1 (1.9%)	
Surgical AEs			.756			.532
Instrumentation malposition	2 (33.3%)	5 (33.3%)		1 (100%)	5 (41.7%)	
Adjacent segment pathology	1 (16.7%)	2 (13.3%)		−(0.0%)	5 (41.7%)	
Wound infection – deep	1 (16.7%)	2 (13.3%)		−(0.0%)	−(0.0%)	
New sensory dysfunction	−(0.0%)	2 (13.3%)		−(0.0%)	−(0.0%)	
New motor dysfunction	1 (16.7%)	1 (6.7%)		−(0.0%)	−(0.0%)	
Recurrence of symptoms	−(0.0%)	1 (6.7%)		−(0.0%)	−(0.0%)	
Pseudarthrosis	−(0.0%)	1 (6.7%)		−(0.0%)	2 (16.7%)	
CSF leak	1 (16.7%)	−(0.0%)		−(0.0%)	−(0.0%)	
Other*	−(0.0%)	1 (5.6%)		−(0.0%)	−(0.0%)	
Consequences of surgical AEs			.706			.786
None	−(0.0%)	2 (13.3%)		−(0.0%)	1 (8.3%)	
Nonoperative inpatient	−(0.0%)	1 (6.7%)		−(0.0%)	−(0.0%)	
Nonoperative outpatient	1 (16.7%)	2 (13.3%)		−(0.0%)	3 (25.0%)	
Reoperation	5 (83.3%)	10 (66.7%)		1 (100%)	8 (66.7%)	
TDN grade of AE			.229			.005
1 (mild AE)	−(0.0%)	1 (6.7%)		−(0.0%)	1 (8.3%)	
2 (mild to moderate AE)	−(0.0%)	−(0.0%)		−(0.0%)	1 (8.3%)	
3 (moderate AE)	5 (83.3%)	14 (93.3%)		−(0.0%)	10 (83.3%)	
4 (severe AE)	1 (16.7%)	−(0.0%)		1 (100%)	−(0.0%)	
5 (death)	−(0.0%)	−(0.0%)		−(0.0%)	−(0.0%)	
Clinical outcome			.392			.528
Excellent	5 (27.8%)	16 (30.2%)		6 (33.3%)	16 (30.2%)	
Good	3 (16.7%)	18 (34.0%)		4 (22.2%)	12 (22.6%)	
Fair	9 (50.0%)	14 (26.4%)		3 (16.7%)	18 (34.0%)	
Poor	1 (5.6%)	4 (7.5%)		4 (22.2%)	6 (11.3%)	
Missing	- (0.0%)	1 (1.9%)		1 (5.6%)	1 (1.9%)	
Working status			.962			.196
Not working (invalidity)	1 (5.6%)	2 (3.8%)		1 (5.6%)	3 (5.7%)	
Not working (retired)	11 (61.1%)	37 (69.8%)		8 (44.4%)	39 (73.6%)	
Not working (sick leave)	3 (16.7%)	7 (13.2%)		1 (5.6%)	1 (1.9%)	
Not working (jobless)	−(0.0%)	−(0.0%)		1 (5.6%)	1 (1.9%)	
Working, full job	2 (11.1%)	4 (7.6%)		3 (16.7%)	6 (11.3%)	
Working, partial job	1 (5.6%)	2 (3.8%)		1 (5.6%)	2 (3.8%)	
Missing	−(0.0%)	1 (1.9%)		3 (16.7%)	1 (1.9%)	
**Total**	**n=18 (100%)**	**n=53 (100%)**		**n=18 (100%)**	**n=53 (100%)**	

⁎ Other listed AEs at 90 days was atraumatic vertebral body fracture of L3 (n=1). Data is presented as count (percent) or mean (standard deviation). TDN, therapy-disability-neurology.

**Table 5:** Information on spino-pelvic parameters in=71 patients with degenerative disc disease undergoing standard TLIF surgery, or TLIF with release of the anterior longitudinal ligament (ALL) between L4 and S1 (transforaminal anterior release = TFAR).

Spino-pelvic parameters	Preoperative			Discharge			90 d postoperative			12 mo postoperative		
	TFAR	Standard TLIF	p-value	TFAR	Standard TLIF	p-value	TFAR	Standard TLIF	p-value	TFAR	Standard TLIF	p-value
PI, in °	56.7 (10.6)	59.2 (11.3)	.399	-	-	-	-	-	-	-	-	-
SS, in °	29.4 (10.6)	40.1 (9.6)	<.001	37.8 (10.3)	38.4 (8.8)	.812	37.3 (10.1)	38.5 (8.7)	.635	37.3 (9.2)	38.5 (8.7)	.626
PT, in °	27.3 (7.6)	19.1 (7.4)	<.001	19.1 (8.9)	19.8 (10.5)	.982	19.4 (8.7)	19.8 (10.4)	.844	19.4 (9.0)	19.8 (10.4)	>.99
Total LL, in °	38.7 (19.2)	54.6 (14.2)	<.001	54.8 (11.1)	52.9 (11.0)	.537	54.9 (12.0)	53.1 (10.2)	.546	56.0 (9.7)	53.1 (10.2)	.297
LL L4S1, in °	21.6 (11.6)	27.1 (11.5)	.084	37.9 (7.9)	23.4 (11.1)	<.001	36.9 (9.0)	23.8 (11.2)	<.001	37.7 (7.8)	24.0 (11.3)	<.001
LLDI, in %	47.5 (47.0)	50.5 (21.7)	.374	70.3 (13.4)	43.4 (17.9)	<.001	69.5 (22.1)	43.9 (18.6)	<.001	68.1 (12.3)	43.4 (19.4)	<.001
Lumbar apex			.129			.458			.312			.340
Disc L2/3	6 (33.3%)	4 (7.6%)		- (0.0%)	−(0.0%)		- (0.0%)	−(0.0%)		- (0.0%)	−(0.0%)	
Vertebra L3	3 (16.7%)	11 (20.7%)		4 (22.2%)	4 (7.6%)		5 (27.8%)	5 (9.4%)		3 (16.7%)	5 (9.4%)	
Disc L3/4	4 (22.2%)	8 (15.1%)		1 (5.6%)	4 (7.6%)		2 (11.1%)	3 (5.7%)		3 (16.7%)	3 (5.7%)	
Vertebra L4	3 (16.7%)	22 (41.5%)		8 (44.4%)	35 (66.0%)		7 (38.9%)	36 (67.9%)		7 (38.9%)	36 (67.9%)	
Disc L4/5	1 (5.6%)	2 (3.8%)		2 (11.1%)	4 (7.6%)		2 (11.1%)	4 (7.6%)		3 (16.7%)	4 (7.6%)	
Vertebra L5	1 (5.6%)	5 (9.4%)		3 (16.7%)	4 (7.6%)		2 (11.1%)	3 (5.7%)		2 (11.1%)	3 (5.7%)	
Disc L5/S1	- (0.0%)	1 (1.9%)		- (0.0%)	1 (1.9%)		0 (0.0%)	1 (1.9%)		- (0.0%)	1 (1.9%)	
Missing	- (0.0%)	- (0.0%)		- (0.0%)	1 (1.9%)		- (0.0%)	1 (1.1%)		- (0.0%)	1 (1.9%)	
	**n=18 (100%)**	**n=53 (100%)**		**n=18 (100%)**	**n=53 (100%)**		**n=18 (100%)**	**n=53 (100%)**		**n=18 (100%)**	**n=53 (100%)**	

Data is presented as count (percent) or mean (standard deviation). LL, lumbar lordosis; LLDI, lumbar lordosis distribution index; PI, pelvic incidence; PT, pelvic tilt; SS, sacral slope.

**Table 6:** Logistic regression analysis of any complication until 12 months, reoperation until 12 months and good or excellent outcome at 12 months postoperative in=71 patients with degenerative disc disease undergoing standard TLIF surgery, or TLIF with release of the anterior longitudinal ligament (ALL) between L4 and S1 (transforaminal anterior release = TFAR).

Outcome of interest	Univariable analysis			Multivariable analysis*		
	OR	95% CI	p-value	OR	95% CI	p-value
Any complication until 12 mo	0.96	0.33–2.83	.951	0.78	0.21–2.94	.717
Any reoperation until 12 mo	0.76	0.25–2.34	.635	0.46	0.11–1.90	.285
Excellent or good outcome at 12 mo	1.22	0.40–3.71	.720	2.01	0.52–7.74	.312

⁎ The multivariable analysis was adjusted for the following variables: age, repeat surgery, segment of TLIF, number of segments included in the fusion. Data is presented as odds ratio (OR) with 95% confidence intervals (CIs).

Stata SE v18 for Mac (StataCorp LLC, College Station, TX [USA]) was used for coding and statistical analysis. p-values of <.05 were considered significant.

## Results

### Study sample

**Fig. 2:**
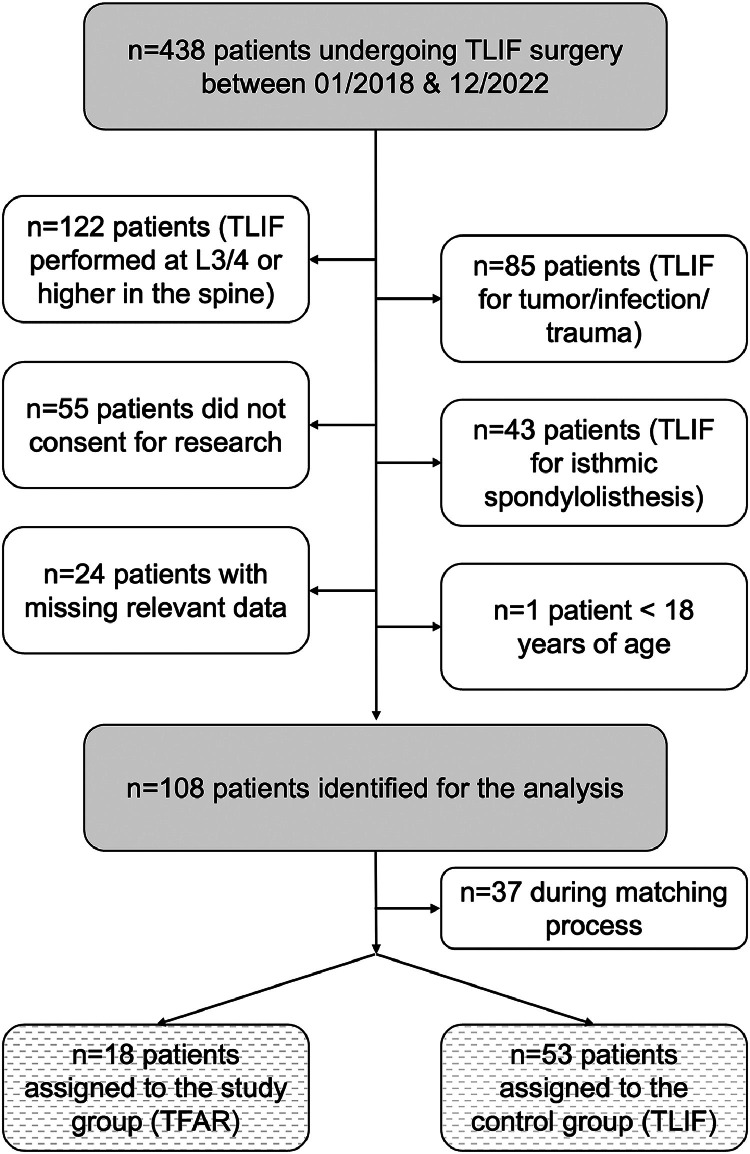
Flow diagram, indicating the reasons for noninclusion of consecutively treated patients undergoing a TLIF procedure at our spine center between 01/2018 and 12/2022. N=108 patients were identified, of which 18 patients underwent a TFAR and were matched in a 1:3 ratio with 53 patients undergoing a standard TLIF procedure.

We identified n=438 patients, who underwent spinal fusion surgery with the TLIF technique during the study period. Of those, n=330 were excluded for the reasons illustrated in Fig. 2. A total of n=108 patients built the final cohort, of which n=37 were excluded during the matching process. Finally, n=18 TFAR patients were matched in a 1:3 ratio with n=53 standard TLIF patients.

Patients undergoing standard TLIF were about 4 years older (p=.074) but sex distribution was similar (Table 1). There were no differences in most of the other baseline demographic information, including, for example, body mass index, smoking status, anesthesiologic risk, main pathology and preoperative symptoms. The preoperative functional status was poor in the vast majority, necessitating the use of weak and strong opioids in a considerable number of patients from both groups. Significant differences were seen in previous surgeries (38.9% in the TFAR group vs. 17.0% in the standard TLIF group) and the level of TLIF (L5/S1 in 66.7% in the TFAR group vs. 28.3% in the standard TLIF group; Table 1).

### Surgical characteristics and complications

Both TFAR and standard TLIFs were conducted by neuro- and orthopedic surgeons alike with similar seniority (Table 2). TFAR was part of a longer construct with anterior-posterior approaches in 22.2% (vs. 9.4% for standard TLIF, p=.159) and multilevel fusion in 27.8% of cases (vs. 5.7% in standard TLIF, p=.034). Hence, both length of the procedure and blood loss were higher and there was more often a need for the use of cell-savers and blood products in patients undergoing TFAR. The rates of intraoperative medical and surgical AEs were not significantly different (Table 2).

### Postoperative course, complications and outcomes

Patients in the TFAR group were more often admitted to the ICU (55.6 vs. 20.7%, p=.033) and their average ICU stay was longer (Table 3). The rates and severities of AEs at time of discharge were similarly high in patients from both groups; medical AEs were more frequent than surgical AEs. Unplanned reoperations had to be performed in 2 patients who underwent the TFAR procedure (11.1%) and in 3 patients from the standard TLIF group (5.7%, p=.435). Discharge disposition and clinical outcomes at discharge were comparable with excellent or good outcomes in 88.9% (TFAR) and 83.1% (standard TLIF, p=.502).

At the 90-day follow-up, we observed similar rates and severities of surgical AEs in both groups (Table 4); an unplanned re-operation was required in 5 patients of the TFAR group (27.8%) and in ten patients of the standard TLIF group (18.9%). At that time, 44.5% of patients in the TFAR group and 64.2% of patients in the standard TLIF group reported an excellent or good outcome, respectively (p=.392).

Outcomes remained similar at the 12-month follow-up, with 5.6% of patients in the TFAR group and 22.6% in the standard TLIF group experiencing an additional surgical AE (p=.213; Table 4). Further revision surgery was required in 1 patient of the TFAR group (6.7%; n=1 missing) and 8 patients of the standard TLIF group (15.1%; n=1 missing). Excellent or good clinical outcomes were reported by 55.5% of the TFAR group and 52.8% of the standard TLIF group (p=.528; Table 4).

### Spino-pelvic parameters and radiological outcome

As shown in Table 5, patients in the TFAR group started on average with 15.9° less total LL, 5.5° less lordosis between L4 and S1, higher PT and lower SS values and a tendency for the lumbar apex to be situated more in the upper lumbar spine, when compared to the standard TLIF group. At time of discharge, most of these differences were not evident anymore. In fact, the lordosis between L4 and S1 was 14.5° higher and the lumbar distribution index was 26.9% higher on average in the TFAR group at discharge. These postoperative results were maintained at the 90-day and 12-month follow-up (Table 5).

### Logistic regression analysis of complications, reoperations and clinical outcomes

In an univariable model, patients in the TFAR group were as likely as patients in the standard TLIF group to experience any complication (OR 0.96, 95% CI 0.33–2.83), any reoperation (OR 0.76, 95% CI 0.25–2.34), or excellent/good outcomes at 12 months postoperatively (OR 1.22, 95% CI 0.40–3.71). In a multivariable logistic regression model, adjusted for patient age, revision surgery, TLIF level and the number of segments included in the fusion construct, the odds for patients in the TFAR group and in the normal TLIF group to experience any complication (aOR 0.78, 95% CI 0.21–2.94), any reoperation (aOR 0.46, 95% CI 0.11–1.90), or excellent/good outcomes at 12 months (aOR 2.01, 95% CI 0.52–7.74) remained similar.

## Discussion

We here set out to analyze the rates of AEs, as well as the clinical and radiological outcomes including spino-pelvic parameters in patients undergoing TFAR between L4 and S1, a powerful technical extension of the TLIF procedure to restore sagittal balance. To better interpret the results, we matched a control group in a 3:1 ratio from the same period, operated by the same surgeons. Essentially, we found that none of the TFAR patients experienced an intra- or postoperative vascular injury, which would be one of the most feared complications of this technique. Altogether, the rates and severities of surgical AEs did not differ much between patients from both groups, and outcomes were comparable. Patients in the TFAR group experienced slightly more often intraoperative medical AEs (not significant), which was likely due to more baseline comorbidities and both longer and more extensive surgical procedures. Compared to the standard TLIF technique, the TFAR technique allowed for a much greater gain in total LL, as well as in lordosis between L4 and S1, which was maintained over the follow-up period up to 12 months.

**Fig. 3:**
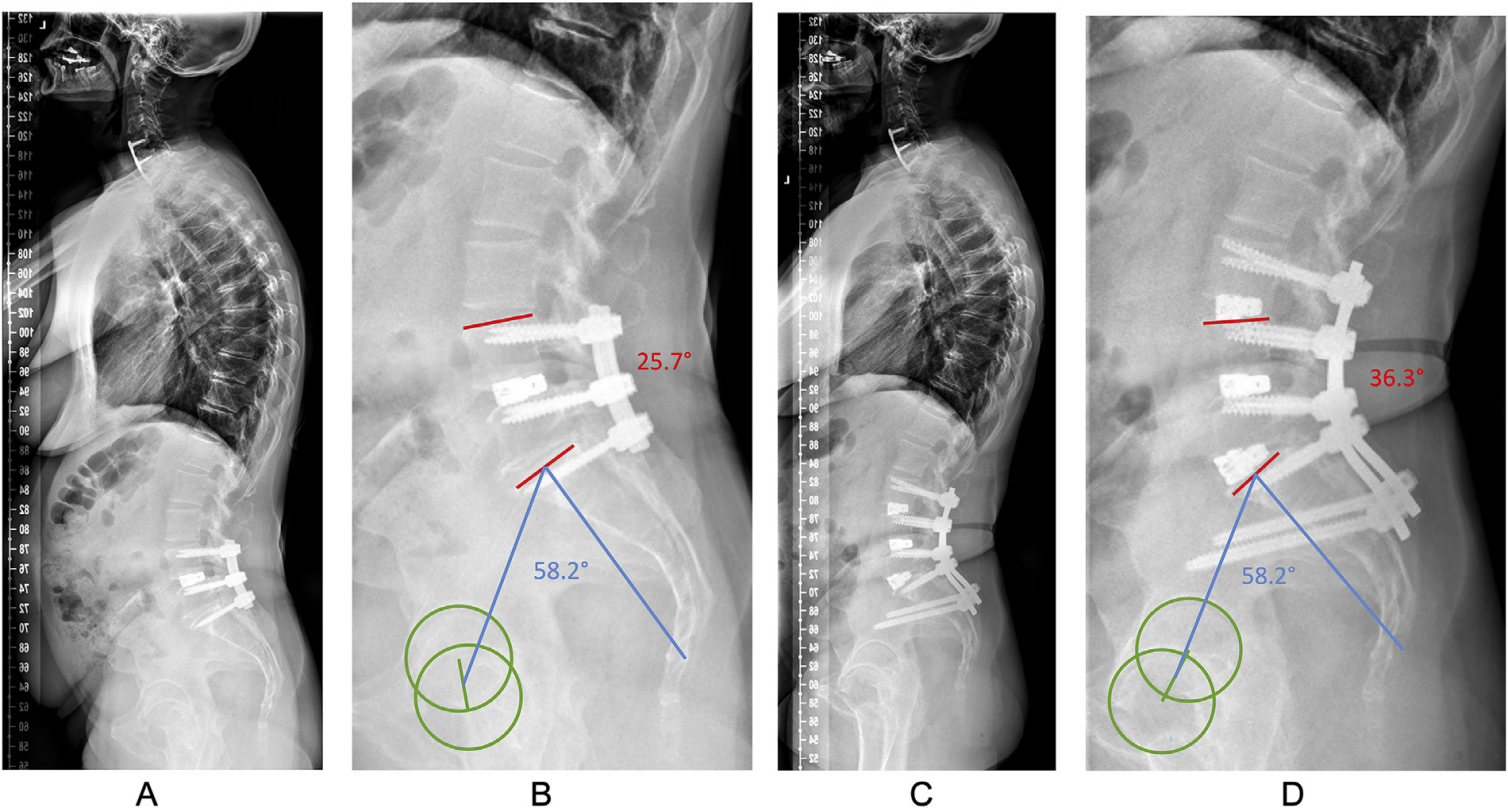
This 58-year-old female with history of 3 prior surgeries between L4 and S1, including decompression and instrumented fusion between L4 and S1 about a year ago presented to our spine center with recurrence of neurogenic claudication, left radicular S1-type pain and sacroiliac joint (SIJ) pain. (A) Lateral standing full-spine x-ray demonstrates a pelvic incidence of 58.2° with a sacral slope of 35°, corresponding to a Roussouly type 3 spinal geometry. Her pelvic tilt was 23°, indicating compensation for lack of lumbar lordosis (LL). (B) Total LL was 56° (mismatch of 2°; ideal: 58°), but lordosis between L4 and S1 was only 25.7° (illustrated in red), corresponding to a lack of 15° between L4 and S1 (ideal: 41°; www.spinebit.io). The lumbar apex was at the disc space L3/4 (ideal: Vertebra L4) and her C7 sagittal vertical axis was 6.2 cm (ideal: <5 cm). (C) We performed a revision procedure with extension of the fusion to L3 and S2Ai, attempting an SIJ-fusion, as well. After removal of the posterior fusion mass and bilateral facetectomy at L5/S1, the segment was remobilized and by controlled release of the ALL by the TFAR technique, sufficient restoration of segmental lordosis was achieved. (C–D) At the 12-month follow-up, total LL was 62°, lordosis between L4 and S1 was 36.3° (illustrated in red), the lumbar apex was corrected to the vertebra L4 and the C7 SVA was close to zero.

The TFAR technique appears as a valuable option in patients with mobile, nonfused spinal segments, where an anterior or lateral approach may not be feasible (e.g., for anatomical reasons; see Fig. 1) or where the workflow can be optimized by doing a single posterior surgery (thereby avoiding posterior-anterior-posterior procedures; see Fig. 3) [3,6]. In our department, we have applied the TFAR technique in both the upper lumbar spine (Th12-L4) and in the lower lumbar spine (between L4 and S1). Herein, we focused on the segments L4/5 and L5/S1, as in these 2 segments usually most of the lordosis needs to be restored. In addition, the TFAR technique is likely most challenging in the lower lumbar segments for several anatomical reasons, including depth of the surgical field, most pronounced degeneration, closest proximity to large blood vessels, and narrowest neural foramen.

Our results stand in contrast with previous studies, such as the one by Han et al., who particularly focused on the levels L1-L3 and did not include a control group [4]. Lattig et al. [6] reported a series of 25 patients with 33 segments, but only in 9 cases the TFAR procedures comprised the segment L4/5. Our results confirm the safety of the TFAR procedure applied in the lower lumbar spine, results which replicate earlier findings reported by Sweet & Sweet [3] and Sabou et al. [5] Our study differs from the report by Sabou et al. [5] in terms of indication by including patients with degenerative pathologies and lack of lordosis but no deformity, in terms of the setting (different hospital and healthcare system), as well as in terms of the surgical technique by concentrating exclusively on the lower lumbar spine and by choosing a TLIF approach with placement of a single, expandable interbody spacer (as contrasted by PLIF with 2 static cages per level). The results in terms of clinical and radiographical outcomes compare well, however, and hence our study can be regarded as an external validation of the previous findings. Moreover, neither our TFAR cohort, nor the patients in the TFAR group reported by previous study groups experienced any vascular or unusual neurological injury [3,5,6].

### Comparative analysis between normal TLIF and TFAR

It should be pointed out that patients, who were selected for the TFAR procedure, had certain characteristics that differ from patients, who were selected for a standard TLIF. Typically, they had a “flat back” deformity with substantial lack of lordosis, particularly in the lower lumbar spine. Moreover, they did not qualify for anterior or lateral interbody fusion, which are our preferred options to restore lordosis, either for anatomical reasons, for workflow considerations or because of the comorbidity burden, making a “posterior-only” approach attractive. Hence, despite similarity of most baseline data, as seen in Tables 1 and 2, these between-group differences render a direct comparison between TFAR and normal TLIF challenging [5].

We noticed longer operation times and more blood loss in the TFAR group, owing primarily to the higher number levels and the extent of the procedure including anterior-posterior fusion approaches (Table 2). Accordingly, patients showed a tendency for more medical complications during anesthesia and had to be admitted more frequently to the ICU for postoperative care (Table 2). Early unplanned reoperations were noticed in both standard TLIF and TFAR patients for typical reasons, including CSF leak, wound infections, and hardware-related complications (Tables 3 and 4), as well as adjacent segment pathology and pseudarthrosis at 12-months follow-up, without significant differences. Despite the ALL release, which renders the operated segment considerably more unstable and may theoretically predispose for nonunion or other hardware-related complications, this could not be observed in our cohort, aligning well with previous studies [[3], [4], [5]].

When critically appraising the clinical outcomes according to the MacNab criteria [12], the rates of “favorable outcomes” – meaning excellent or good – were highest at discharge, lowest around the 90-day follow-up (likely influenced by AEs and reoperations in this period) and improved over the postoperative period until the 12-month follow-up. Our hospital serves as tertiary reference center for the greater area of Eastern Switzerland, hence gathering a relatively ill patient population in advanced age, characterized by significant comorbidities, poor bone quality and frequent prior attempted fusion surgery, which suffer enormously but are rejected by the surrounding private and smaller public hospitals. Considering these circumstances, the rates of 45% to 90% of favorable outcome appears acceptable. Like Sabou et al. [5], we also did not notice differences in outcomes between groups.

### Spino-pelvic parameters

Sweet and Sweet stated in their paper that with ALL release, the restoration of SL was simplified in comparison to a pedicle subtraction osteotomy (PSO) [3]. In contrast to these authors, our series did not include many patients with fixed sagittal imbalance, as we do prefer PSOs for correction of the fused spine. We did apply the TFAR technique in patients with prior posterior fusion, where the disc space itself could easily be remobilized after resection of the posterior fusion mass and bilateral facets, however (compare Fig. 3).

Comparing the spino-pelvic parameters before and after the procedure, patients in the TFAR group started with an average of 38.7° of total LL and 21.6° of lordosis between L4 and S1. The distribution of lordosis across the lumbar spine was distorted (lumbar lordosis distribution index (LLDI) 47.5% on average) and the lumbar apex too high in many of the patients (Table 5). By restoring the LL in the lower lumbar segments using the TFAR technique, we were not only able to regain total LL and lordosis between L4 and S1, but also to increase the LLDI to approximately 70% and lower the apex to lower segments. At discharge, the net gain in total lordosis was 16.1° in the TFAR group, whereas the standard TLIF group lost about 1.7° of lordosis. Between L4 and S1, the net gain in the TFAR group was 16.3°, whereas the standard TLIF group lost about 3.7° of lordosis. These measurements persisted over the follow-up period until 90 days and 12 months postoperatively (Table 5).

Sweet and Sweet used the TFAR technique with a quite aggressive open wedge correction technique to achieve a maximum of SL [3]. The authors reported an average increase in lordosis of 36.5° (range 24°–56°) in the cohort of patients with fixed sagittal deformity and 22.9° (range 13°–32°) in the segmental kyphosis group. Unlike us, these authors placed the interbody cage in the posterior third of the disc space to use it as a pivot point for maximum lordosis restoration by posterior rod compression. They also used recombinant bone morphogenetic protein 2 (rhBMP-2) in their patients to accelerate bone formation and enable fusion in a considerably destabilized spinal segment. We did not use rhBMP-2 in any case and corrected only as much as was needed for each segment in the individual patient, determined by thorough surgical planning using a self-developed online calculator (www.spinebit.io) and planning software (www.surgimap.com). Hence, more lordosis could have been achieved in our patients, but it was not necessary and would have led to over-correction, which in turn could have detrimental effects in terms of mechanical complications [13,14].

Our results indicate globally balanced spines postoperatively (Table 5), and they align well with the findings of Sabou et al., whose analysis also comprised a control group of patients undergoing standard TLIF or PLIF [5]. These authors reported a pre to postoperative change in total LL of 25.2° (TFAR) versus 15.2° (standard TLIF), and in lordosis between L4 and S1 of 11.0° to 23.7° (TFAR) versus −0.9° to 4.9° (standard TLIF). Albeit applied to the upper lumbar spine [4], Han et al. [4] reported a segmental gain of lordosis of approximately 16°, which is also in agreement with our measurements. In their series of eleven patients with TFAR, the authors achieved a total gain in LL of 28.6°, with the TFAR level accounting for about 49° of the total correction of sagittal imbalance. Finally, Lattig et al. [6] reported an average SL of 11.4° (range 5°–29°) in their TFAR series, mostly comprising the segments L1–L4 (72.7%). In ten of their patients with additional, extended posterior column osteotomies, the gained mean SL increased to 19° (range 14°–29°). From our own results and review of the literature it can be concluded that through the use of the TFAR technique, adequate gain of SL can be achieved to a degree that comes close to the powerful techniques of 3-column osteotomies [6,15]. In direct comparison, TFAR can restore considerably more SL than a standard TLIF.

### Strengths and limitations

While there have been 4 previous publications discussing the TFAR procedure [3,4,6], only one has directly compared TFAR to a conventional TLIF procedure [5]. Our goal was to fill the existing knowledge gaps and publish our outcome data of a series of patients undergoing this relatively novel surgical technique. While our study was retrospective in nature and the sample size of TFAR procedures performed at the levels L4-S1 was moderate, the data collected were comprehensive and the burden of missing data was low.

The main weakness of this study is that the indications for a TFAR procedure typically differ from those for a standard TLIF. In fact, the indications for a TFAR resemble more the indications for an anterior lumbar interbody fusion (ALIF). However, as complications of the ALIF approach are often approach-related, it did not seem appropriate to choose an ALIF cohort for comparison. Including a matched cohort of TLIF patients without ALL release, operated during the same period by the same surgeons, provides insights regarding the comparative risk profile of both techniques. As some between-group differences persisted despite matching, we included a multivariable logistic regression model for 3 main outcomes (AEs, reoperations, and clinical outcome at 12 months), adjusted for the main confounders. Another weakness of this study is the lack of standardized PROMS [16], which were only introduced in our center in the beginning of 2022 and are hence not available for the majority of included patients. Also, some of the TFAR procedures were conducted with 2 experienced surgeons [17], whereas standard TLIF procedures were usually performed by a single attending and a resident or fellow, which may have influenced some of the results. Lastly, as the sample size was limited to n=18 patients with TFAR, some of the calculations may have been underpowered to exclude significant differences and did not allow for selecting a more homogenous cohort (e.g., only patients with a degenerative or deformity indication for surgery).

## Conclusions

In this study of n=18 patients treated by TFAR between L4 and S1, this technique appeared to have a safety profile which is likely comparable to the standard TLIF procedure, but it allows for a much greater restoration of lumbar lordosis at L4-S1. We suggest considering the TFAR technique in selected patients with sagittal imbalance and mobile segments, especially if not suitable for ALIF or LLIF with ALL release.

## Funding statement

No funding was received for this research.

## Declaration of generative AI and AI-assisted technologies in the writing process

No AI or AI-assisted technologies were used for the writing process of this article.

## Declaration of competing interests

The authors report no conflicts of interest.

## Appendix Supplementary materials

Image, video 1

Image, video 2

Image, application 3
