# Supplementary material for: Transforaminal lumbar interbody fusion with or without release of the anterior longitudinal ligament: A single-center, retrospective observational cohort study
Source: N Am Spine Soc J. 2024 Jul 29;19:100533. doi: 10.1016/j.xnsj.2024.100533 (PMC11385389; doi:10.1016/j.xnsj.2024.100533)
Supplement: Supplementary file 3 [file mmc3.docx]

**Video legends**

**Video 1**: The video demonstrates how the anterior longitudinal ligament (ALL) is initially opened in a safe region without proximity to blood vessels, using a 4mm chisel and gentle taps of the palm. Then, a 2mm Kerrison punch is used to resect the ALL further. This is usually done from both patient sides.

**Video 2**: After resection of the anterior longitudinal ligament (ALL), a thorough inspection is conducted to visualize and palpate, if necessary, the retroperitoneal fat in order to make sure the ALL release is sufficient.
